# Clarence Visick

**Published:** 1910-07-09

**Authors:** 


On June 22 the death from pneumonia occurred at Malaga of Clarence Visick, M.R.C.S., L.R.C.P., aged sixty-eight. The deceased physician had lived and practised in Spain probably longer than any other English medical man, and was well known to every traveller whose journeys had taken him to the increasingly popular resort which Mr. Visick had made his home. He had been resident medical officer at St. George's Hospital before he went into exile in Spain.

